# Efficacy of the 20-minute whole blood clotting test (WBCT20) in the diagnosis of coagulation alteration related to snakebites in a Western Brazilian Amazon hospital

**DOI:** 10.1590/0037-8682-0091-2021

**Published:** 2021-06-02

**Authors:** Tamires Nascimento da Costa, Ageane Mota da Silva, Rodrigo Medeiros de Souza, Wuelton Marcelo Monteiro, Paulo Sérgio Bernarde

**Affiliations:** 1 Universidade Federal do Acre, Campus Floresta, Laboratório de Herpetologia, Cruzeiro do Sul, AC, Brasil.; 2 Universidade Federal do Acre, Programa de Pós-Graduação Stricto Sensu em Ciências da Saúde na Amazônia Ocidental, Rio Branco, AC, Brasil.; 3 Instituto Federal do Acre, Campus de Cruzeiro do Sul, Cruzeiro do Sul, Acre, Brasil.; 4 Universidade Federal do Acre, Campus Floresta, Laboratório de Microbiologia, Imunologia e Parasitologia, Cruzeiro do Sul, AC, Brasil.; 5 Universidade do Estado do Amazonas, Manaus, AM, Brasil.; 6 Fundação de Medicina Tropical Dr. Heitor Vieira Dourado, Manaus, AM, Brasil.

**Keywords:** Snake bites, Blood coagulation tests, Venoms, Bothrops

## Abstract

**Introduction:**

The efficacy of 20-minute whole blood clotting (WBCT20) and the Lee-White clotting time (LWCT) tests in diagnosing coagulation alterations from snakebites were compared.

**Methods:**

We evaluated 89 snakebite cases treated at the *Hospital Regional do Juruá em Cruzeiro do Sul*, Acre, Brazil.

**Results:**

WBCT20 results were normal in 33.7% and unclottable in 66.3% of cases, while LWCT results were normal in 23.6% and altered (prolonged or unclottable) in 76.4% of cases, with no significant differences.

**Conclusions:**

The WBCT20 is important for rapidly diagnosing coagulation alterations from snakebites. Furthermore, it is efficient, inexpensive, and can be deployed in isolated hospitals.

Snakebites are a challenge for the health of tropical and subtropical populations in areas that have limited resources for the clinical management of patients^1^. The World Health Organization and several other institutions have indicated the use of the 20-minute whole blood clotting test (WBCT20) as an indicator of systemic envenoming and for early treatment of ophidism^1^. The increased coagulation time can confirm the diagnosis of snakebite by demonstrating coagulopathy and the consequential systemic effects of envenoming and can also be used to evaluate the successfulness of serotherapy through patient monitoring until the reversal of incoagulability^2^. 

Bothropic envenoming corresponds to most snakebite cases in Brazil and can promote coagulopathy and cause hemorrhagic manifestations^3^ ^,^ ^4^. This action stems from the hydrolysis of fibrinogen into fibrin and procoagulant activity that activates coagulation factors II and X^4^, thus making the blood unclottable. 

In Brazil, the Ministry of Health^5^ recommends using the Lee-White blood clotting test (LWCT) to aid in the diagnosis of snakebites and to evaluate the effectiveness of antivenom. Despite being a simple test, not all hospitals located in remote regions of the Amazon include laboratories or conditions for carrying out the test. Although the WBCT20 is used in a complementary way to evaluate the coagulant effect of snake venom that makes the blood uncoagulable^1^, its accuracy must be evaluated by comparing it with other alternatives such as the LWCT, plasma fibrinogen levels, and the international nationalized ratio. Therefore, this study evaluated the sensitivity of the WBCT20 in the confirmation of snakebite diagnoses in a hospital in the Western Brazilian Amazon.

In this study, we evaluated snakebite cases treated at the *Hospital Regional do Juruá* (HRJ) in the municipality of Cruzeiro do Sul, Acre, Brazil, from July 1, 2017 to June 30, 2018. Clinical and epidemiological information from this study has been previously published^6^. During this period, there were 133 cases of snakebites; the WBCT20 was performed in 99 and the LWCT in 113 cases (Figure 1). In 89 patients, both blood clotting tests were performed. In all snakebite cases, the envenoming was confirmed by the clinical diagnosis associated with alterations in laboratory examination results, enzyme-linked immunosorbent assays for the identification of circulating bothropic toxins, and/or the identification of the snake if it was taken to the hospital or photographed by the patient or their companion (see details of the methodology in Mota-da-Silva et al^6^ ^,^ ^7^). This study was approved by the Human Research Ethics Committee of the Tropical Medicine Foundation Doctor Heitor Vieira Dourado (opinion number 2,084,630) and was conducted in accordance with the principles of the 1964 Declaration of Helsinki and its later amendments.

**FIGURE 1:**
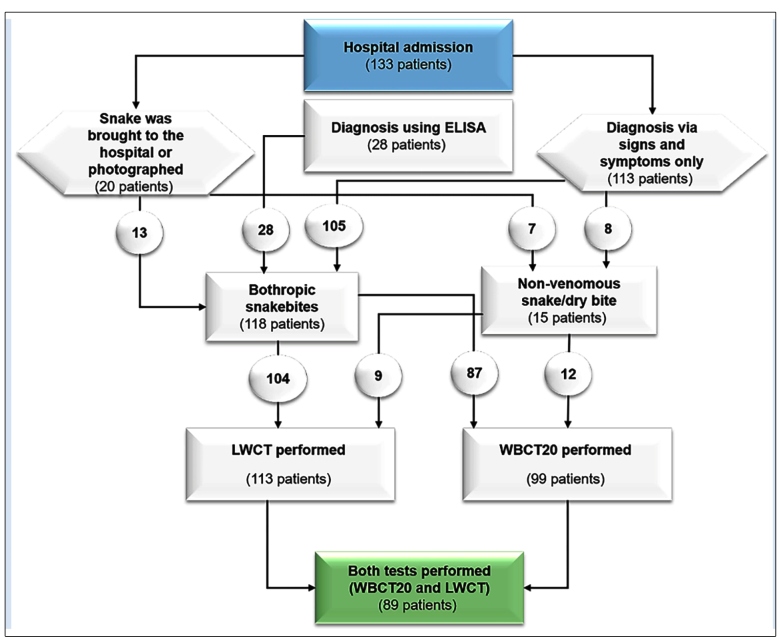
Flowchart showing the stages of patient selection for the study. **ELISA:** enzyme-linked immunosorbent assay; **LWCT:** Lee-White clotting time test; **WBCT20:** 20-minute whole blood clotting time test.

On admission to the hospital, the WBCT20 was performed by the researchers of this study. For this test, 2 ml of venous blood was collected in a clean, new, dry, glass test tube, which was left upright, motionless, and at room temperature for 20 minutes. The test was considered positive when, after 20 min, the blood was still liquid (uncoagulated) and negative when it was semisolid (coagulated)^8^ (Figure 2A and 2B). The LWCT was performed before, during, or after serotherapy according to the hospital protocol (Table 1). Venous blood samples were obtained and immediately placed in 2 glass tubes (13 × 75 mm). The tubes were placed in a water bath at 37 °C for 5 min. The time of blood collection was recorded. After 5 min, the sample was checked for clot formation by tilting the tube to 90 degrees. The time taken for clot formation indicated the whole blood clotting time^9^. Statistical analyses were performed using the SPSS software, version 25.0 (IBM, Corp.; Armonk, NY, USA). Chi-squared tests were used to test associations at a 5% level of significance.

**FIGURE 2:**
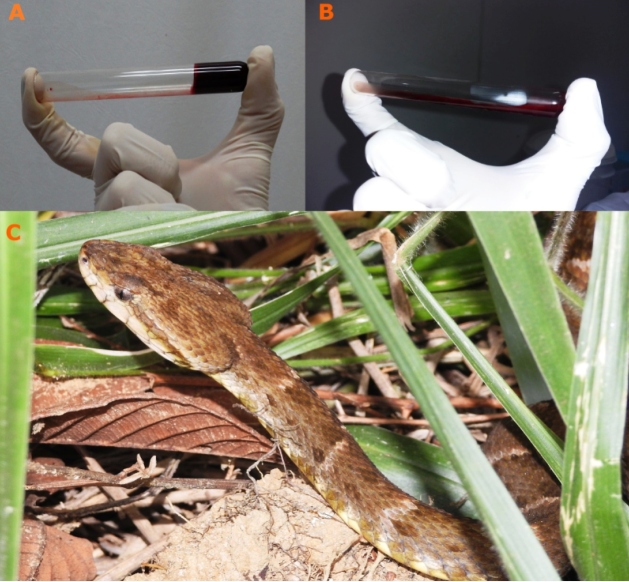
**A)** Negative WBCT20 result (clotted blood); **B)** Positive WBCT20 result (unclottable blood); **C)** Common lancehead (*Bothrops atrox*), the snake responsible for most snakebites in the region. WBCT20: 20-minute whole blood clotting test.

We analyzed the coagulation tests of 89 patients who were victims of snakebites; 81 (91%) of these were caused by the *Bothrops* genus of snakes (Figure 2C) while the rest were attributed to nonvenomous snakes or dry bites. The WBCT20 results were normal and unclottable in 30 (33.7%) and 59 (66.3%) patients, respectively. The LWCT results were normal in 21 (23.6%) and abnormal in 68 (76.4%) cases, of which 58 (65.2%) had prolonged results and 10 (11.2%) were unclottable (Table 1). The test results were concordant in 60 (67.4%) patients: 11 (12.4%) were normal and 49 (51.1%) were abnormal in both tests. There were no significant differences between the WBCT20 and LWCT coagulation test results (chi-squared = 4.311, *p* = 0.116). 

**TABLE 1:** Group of 89 snakebite patients treated at HRJ who received WBCT20 and LWCT blood coagulation tests

		WBCT20				LWCT				
Variable	Normal	%	Unclottable	%	Normal	%	Prolonged	%	Unclottable	%
**Type of snakebite**										
Bothropic (81)	26	32.1	55	67.9	16	19.8	55	67.9	10	12.3
Non-venomous/dry bites (8)	4	50	4	50	5	62.5	3	37.5	-	
**Time between snakebite and WBCT20 collection**										
Up to 1 hour (15)	6	40	9	60	-		-		-	
2 to 6 hours (43)	14	32.6	29	67.4	-		-		-	
7 to 24 hours (27)	8	29.6	19	70.4	-		-		-	
After 24 hours (4)	2	50	2	50	-		-		-	
**Time between accident and LWCT collection**										
Up to 1 hour (2)	-		-		1	50	1	50		
2 to 6 hours (39)	-		-		9	23.1	24	61.5	6	15.4
7 to 24 hours (32)	-		-		9	28.1	20	62.5	3	9.4
After 24 hours (6)	-		-		1	16.7	4	66.7	1	16.7
**LWCT collection time**										
Before serotherapy (22)	-		-		5	22.7	14	63.6	3	13.6
During serotherapy (14)	-		-		3	21.4	9	64.3	2	14.3
After serotherapy (37)	-		-		8	21.6	24	64.9	5	13.5

Abbreviations: **HRJ:** Hospital Regional do Juruá em Cruzeiro do Sul, Acre, Brazil; **LWCT:** Lee-White clotting time test; **WBCT20:** 20-minute whole blood clotting time test.

The WBCT20 in this study proved to be an efficient method for determining the presence of systemic envenoming since the results were consistent with the LWCT in 67.4% of patients. Our results corroborate the findings of other studies^1^ ^,^ ^2^ in which WBCT20 was found to be a predictor of the systemic alterations caused by snakebite envenoming and aided in the recommendation for immediate antivenom therapy, thus preventing patients from being exposed to the adverse effects of antibiotics or unnecessary serotherapy. Some cases of altered coagulation time in patients either bitten by nonvenomous snakes or those with dry bites are probably due to the fact that bites from some snakes of the Dipsadidae family (e.g., *Helicops angulatus*) can cause this type of coagulopathy^10^. 

Among the 59 (66.3%) patients with a positive WBCT20 result, 49 (55.1%) also had altered LWCT results. In 19 (21.3%) cases, the WBCT20 results were normal, and the second test showed a change in blood coagulation. In 4 (4.5%) of these patients, the WBCT20 sample was collected between 30 min and 1 h after the snakebite, therefore it is unlikely that coagulopathy could have been established at the time of blood collection, especially in cases where the test was performed between 30 and 40 min after envenoming.

Ratnayake et al.^11^ observed that for the WBCT20 to present good sensitivity, it is essential to use a standard 5 ml tube with only 1 ml of blood. In this study, 2 ml of blood was used for the test, and this may explain the false negatives. Another possible cause is reading the test results at 20 and 30 minutes, although in some cases the interpretation of the results is different. A study carried out in Benin recommends that reading the results at 30 min reduces the proportion of false negatives^12^. In places where this method is proposed, the use of clean, new, glass tubes is essential, since polypropylene and polyethylene containers prolong coagulation time and can cause incorrect interpretations^13^.

The differences in blood clotting times between the LWCT (76.4%) and WBCT20 (66.3%) can be explained by the fact that the LWCT sample was generally collected during or after the administration of antivenom (57.3% of cases) and coagulability might have already been restored. Sousa et al.^4^ observed that the LWCT produced false negatives in their study, which may occur in cases of mild coagulopathy in which the LWCT may present normal results even with low fibrinogen concentrations, in addition to other factors related to the methods used by hospital staff. 

The distance that victims who reside in isolated areas need to travel in order to receive hospital medical care increases the time between the bite and the occurrence of initial treatment and greatly affects the patient's prognosis^6^ ^,^ ^7^. In many cases, patients come from rural areas of municipalities that do not have hospitals with the necessary infrastructure to perform more sophisticated coagulation tests. In Thailand, Wongkrajang et al.^14^ concluded that the WBCT20 can be promoted as a standard coagulation test in places with no other available methods. However, it is emphasized that for this implementation, it is necessary to standardize the test with the use of appropriate materials and staff training.

Benjamin et al.^12^ raised a question that needs to be better investigated regarding the link between the presence of local edema and a possible recurrence of envenoming after generalized edema has disappeared. In this theory, compartments with venom become trapped in edematous regions, and after the swelling has decreased, the amount of venom in the bloodstream could increase again. In accordance with this hypothesis, it would be important to repeat the coagulation test after the reduction of edema to verify a possible recurrence of envenoming and aid in therapeutic measures. Rajeswari and Suneetha^15^ observed that the WBCT20 was quite effective for determining if additional doses of antivenom should be administered and contributed to an improvement in patients' renal function, thereby decreasing the need for hemodialysis.

The WBCT20 has proven to be an important tool for assisting in the rapid diagnosis of bothropic envenoming. The absence of coagulopathy does not rule out the possibility of envenoming since not all cases of bothropic snakebites cause this hemostatic disorder^2^ ^,^ ^4^. In both situations, it is necessary to evaluate the characteristic signs and symptoms for the diagnosis. As suggested by Sousa et al.^4^ for the LWCT, we also suggest taking precautions to avoid delayed serotherapy in cases of false-negative results or the unnecessary use of serotherapy in false-positive patients. More in-depth studies (e.g., regarding fibrinogen consumption) are essential to better evaluate the sensitivity and efficiency of the WBCT20 for the diagnosis of snakebites and to monitor the restoration of blood coagulability after serotherapy. 

The WBCT20 may be an efficient and inexpensive method that can be employed in many hospitals, or even in basic health units, in isolated locations of the Brazilian Amazon and could contribute to the confirmation of snakebite envenoming, especially in rural areas where these cases occur most frequently and many facilities do not have laboratories equipped with the material resources to perform coagulation tests. However, standardization of the method and proper training of professionals are necessary to avoid misinterpretations.
